# Contribution of Psychosocial Factors to the Association between Socioeconomic Position and Takeaway Food Consumption

**DOI:** 10.1371/journal.pone.0108799

**Published:** 2014-09-30

**Authors:** Kyoko Miura, Gavin Turrell

**Affiliations:** 1 School of Public Health and Social Work, Faculty of Health, Queensland University of Technology, Kelvin Grove, Queensland, Australia; 2 Cancer and Population Studies Group, QIMR Berghofer Medical Research Institute, Herston, Queensland, Australia; Kyushu University Faculty of Medical Science, Japan

## Abstract

**Objective:**

To examine whether psychosocial factors mediate (explain) the association between socioeconomic position and takeaway food consumption.

**Design:**

A cross-sectional postal survey conducted in 2009.

**Setting:**

Participants reported their usual consumption of 22 takeaway food items, and these were grouped into a “healthy” and “less healthy” index based on each items' nutritional properties. Principal Components Analysis was used to derive three psychosocial scales that measured beliefs about the relationship between diet and health (α = 0.73), and perceptions about the value (α = 0.79) and pleasure (α = 0.61) of takeaway food. A nutrition knowledge index was also used. Socioeconomic position was measured by highest attained education level.

**Subjects:**

Randomly selected adults (n = 1,500) aged between 25–64 years in Brisbane, Australia (response rate  =  63.7%, N = 903).

**Results:**

Compared with those with a bachelor degree or higher, participants with a diploma level of education were more likely to consume “healthy” takeaway food (p = 0.023) whereas the least educated (high school only) were more likely to consume “less healthy” choices (p = 0.002). The least educated were less likely to believe in a relationship between diet and health (p<0.001), and more likely to have lower nutritional knowledge compared with their highly educated counterparts (p<0.001). Education differences in beliefs about the relationship between diet and health partly and significantly mediated the association between education and “healthy” takeaway food consumption. Diet- and health-related beliefs and nutritional knowledge partly and significantly mediated the education differences in “less healthy” takeaway food consumption.

**Conclusions:**

Interventions that target beliefs about the relationship between diet and health, and nutritional knowledge may reduce socioeconomic differences in takeaway food consumption, particularly for “less healthy” options.

## Introduction

There are well-established socioeconomic inequalities in health [1], [2]. Diet is a major contributing factor to the poor health of socioeconomically disadvantaged groups [3], [4], and the dietary patterns of these groups are less likely to be consistent with dietary recommendations [5], [6]. In this paper, we examine the relationship between socioeconomic position (SEP) and the consumption of “takeaway food”, here defined as foods or meals that are pre-prepared commercially and require no further preparation by the consumer, and can be consumed immediately after purchase. Takeaway foods include “fast-food” and “convenience food”, and these types of food are often associated with diets that are high in energy and total fat, and low in essential nutrients (e.g. vitamin A and C); they are also associated with negative health-related outcomes including weight gain [11]. Previous studies have reported that socioeconomically disadvantaged groups are more likely than their advantaged counterparts to consume or purchase takeaway food [7]–[10] and this might partly explain why disadvantaged groups have a higher prevalence of overweight and obesity [11] and why they experience higher rates of mortality and morbidity for diet-related chronic disease [3], [4], [12], [13]. To date, however, our understanding of why socioeconomic group differ in their consumption of takeaway food is limited. It has been suggested that psychosocial factors might contribute to this association [14]–[17]; however, no known study has investigated this issue.

We define “psychosocial” as pertaining to the influence of social and structural factors (e.g. SEP) on an individual's psychological disposition (e.g. beliefs, attitudes, perceptions) such that their interrelation shapes and circumscribes behaviour, and ultimately, health. In the context of this study we focus on four individual-level psychological factors—knowledge, beliefs, preferences, and perceptions—and conceptualise these as potentially mediating the effects of SEP on takeaway food consumption.

Nutritional knowledge and health beliefs may partially explain socioeconomic differences in takeaway food consumption. Socioeconomically disadvantaged groups are more likely to have low levels of nutritional knowledge and are less likely to believe in the relationship between diet and health compared with advantaged groups [18]–[20] and these factors have been associated with less healthy dietary intakes [21]–[23], including frequent fast-food consumption [24].

Taste or food preference is one of the most influential determinants of food choice [25] and is also an important reason for the consumption of fast-food [26]. Hence, taste is also likely to be an important determinant of takeaway food consumption. Food preference may also vary across different socioeconomic groups. Lower income households have been reported to be more likely than their affluent counterparts to dislike foods that were consistent with dietary guideline recommendations [27]. This finding suggests that socioeconomically disadvantaged groups may prefer the taste of takeaway foods more than advantaged groups, and this may contribute to the more frequent takeaway food consumption among disadvantaged groups.

The perception of takeaway food as value for money may be an important factor influencing the decision to consume takeaway food [7], [28], [29]. Consumers may perceive takeaway food as worth purchasing if they can trade-off the expense for a reduction of time and effort for meal planning, preparation and cleaning up [30]. A previous study found that frequent consumers of takeaway food were more likely to report that convenience food (including takeaway foods) represented value for money compared with those who consumed takeaway food less regularly [28].

In health research, the three most commonly used individual-level measures of SEP are education, occupation, and income [31]. Given that these indicators are only moderately correlated [32], it is likely that they capture different dimensions of the socioeconomic construct and probably reflect distinct aetiological pathways between socioeconomic circumstances and takeaway food consumption [33]. In this paper, we use education as our socioeconomic indicator as it is arguably the most conceptually meaningful socioeconomic determinant of an individual's psychological disposition towards food choice. Education reflects knowledge and skills (cognitive capacities) attained through formal learning (e.g. school, university) and lived experience, and these are likely to be important factors that shape people's dietary beliefs, perceptions and preferences, and influence the acquisition of information and knowledge about healthy dietary behaviours.

While earlier research has investigated psychosocial influences on takeaway or fast-food consumption [7], [28], [34]–[37], no known studies have assessed the contribution of psychosocial factors to different types of takeaway food defined as “healthy” or “less healthy”: the former may be influenced by different psychosocial factors compared with the latter. For example, consumption of “healthy” takeaway items, such as sushi, may be driven by strong health beliefs or high nutritional knowledge whereas taste may be the dominant reason for the consumption of “less healthy” items such as fried potato chips. The aims of this study are to determine whether psychosocial factors mediate (explain) the association between education and the types of takeaway food consumed among adults.

## Methods

### Study Participants

This cross-sectional study was conducted in the Brisbane metropolitan area (Australia) between July and September 2009. A total of 1,500 adults aged between 25–64 years were randomly selected from the electoral roll of the Brisbane statistical subdivision. Data were collected by a postal survey [38] that asked about usual takeaway food consumption, a range of psychosocial factors that may influence consumption, and socio-demographic characteristics. A total of 903 participants completed the survey (response rate 63.7%). For the purpose of this paper, participants who reported never consuming takeaway food in the previous 12 months (n = 19, 2.1%) were excluded from the analyses. Ethical approval for the study was granted by the Queensland University of Technology Human Research Ethics Committee (ID 0900000445).

### Measures

#### Socioeconomic position and covariates

Education was used as the socioeconomic measure and ascertained by the highest completed qualification. Participant's education was coded as: 1) bachelor degree or higher (latter includes graduate diploma, graduate certificate, and postgraduate degree); 2) diploma (includes associate degree which is generally not a university-level education in Australia); 3) vocational (trade or business certificate); and 4) no post-school qualifications. Covariates used in the multivariable analyses were age (continuous) and sex.

#### Psychosocial factors (mediators)

Based on previous research, participants were asked a range of questions about psychosocial factors that may influence takeaway food consumption [8], [24], [26].

Belief about the diet-health relationship, perceived values of takeaway food, and takeaway food as pleasure: Participants were asked to indicate their level of agreement (1 = strongly disagree, 5 = strongly agree) with the following items: “Eating a diet that is high in fat is a threat to my health”, “Being 10 kg or more overweight is a threat to my health” [8], “What you eat can affect your chance of getting cancer or heart disease” [24], “Takeaway foods are value for money”, “Takeaway foods are inexpensive”, “Takeaway food is fun and entertaining”, “Takeaway food is a treat for myself”, “Takeaway foods are tasty” [26], and “It is cheaper for me to buy takeaway foods than to cook for myself” [39]. These nine items were included in a Principal Component Analysis to determine if there was an underlying structure to the beliefs and perceptions data. Using Varimax rotation and eigenvalue criteria ≥1.0, three components were identified and subsequently interpreted as “diet-health belief and weight concern”, “perceived value of takeaway food” and “takeaway foods as pleasure” (Table 1). These three components had eigenvalues of 2.6, 1.9, and 1.3 respectively, they accounted for 28.9%, 21.1%, and 14.5% of the total variance, and their cumulative contribution was 64.6%. Standardised scoring coefficients were calculated for the items forming the three components and these were used to derive factor scales for each of the constructs.

**Table 1:** Beliefs and perceptions about diet, health, and takeaway food: results of a Principal Components Analysis.

(N = 801)	Retained components (loadings)		
	1	2	3
**Belief about the diet-health relationship** †			
Eating a diet that is high in fat is a threat to my health	**0.74**	−0.05	0.04
What you eat can affect your chance of getting cancer or heart disease	**0.83**	−0.03	−0.03
Being 10 kg or more overweight is a threat to my health	**0.84**	−0.10	0.00
Cronbach's Alpha	**0.73**		
**Perceived value of takeaway food** †			
Takeaway foods are value for money	−0.03	**0.83**	0.19
Takeaway foods are inexpensive	−0.03	**0.86**	0.07
It is cheaper for me to buy takeaway foods than to cook for myself	−0.15	**0.79**	0.08
Cronbach's Alpha		**0.79**	
**Takeaway foods as pleasure** †			
Takeaway food is fun and entertaining	0.02	0.23	**0.72**
Takeaway food is a “treat” for myself	0.01	−0.05	**0.80**
Takeaway food is tasty	−0.01	0.15	**0.70**
Cronbach's Alpha			**0.61**

† Response options for each item range from 1  =  Strongly Disagree to 5  =  Strongly Agree.

Nutritional knowledge: A 20-item nutritional knowledge measure was adapted from a previous study [40]. These items cover knowledge about the nutrient content of various foods, the relationship between nutrition and health, and dietary recommendations. Participants had three response options for each statement: “true”, “false”, or “not sure”. A score was calculated to determine each participant's general nutritional knowledge according to their correct answers to the statements: a score of 1 was assigned when the response was correct, and if the response was incorrect or “not sure”, the score was 0. A nutritional knowledge index was constructed by summing all items and the index ranged from 0–20 (mean 17.5, SD 2.8; median 18.0). The intra-class correlation coefficient (ICC) of the nutritional knowledge index was 0.91 (95% CI 0.82, 0.95) which is interpreted as “almost perfect” reliability [41].

Takeaway food consumption: As part of the questionnaire, participants were asked *“In the last 12 months, did you eat any takeaway food”?* The response options were “Never”, “Rarely”, “Less than once a month”, “1–3 times per month”, “Once per week”, “2–4 times per week”, “5–6 times per week” and “Once per day”. Respondents who indicated “Never” (n = 19) were directed to a section of the questionnaire which explored possible reasons for not consuming takeaway food: these respondents are not included in this present study. Those who reported consuming takeaway food in the last 12 months were then asked to indicate how often they had consumed each of 22 specific takeaway items. The response options for this question were: “Never or rarely”, “Less than once a month”, “1–3 times per month”, “Once per week”, “2–4 times per week”, “5–6 times per week”, “Once per day or more”. The 22 takeaway foods were kebab, sandwiches, fried rice, pasta, Asian-style noodles, sushi, salad, diet soft drink, fruit/vegetable juices, fried potato chips, hamburger, pizza, savoury pies, fried fish/seafood, fried chicken, fried dim-sum, curry, cakes, non-diet soft drink, thick/milk shake, flavoured milk, and ice-cream. At the time the questionnaire was administered, these were the most frequently consumed takeaway items in Australia [9].

Each of the 22 items was categorised as either “healthy” or “less healthy” based on the Australian Guide to Healthy Eating (AGHE) [42] which classifies food into five core food groups and an “extra” food group. The “extra” foods (e.g. cakes and deep-fried takeaway foods) are a non-essential part of a diet and are typically high in fat, sodium, or sugar. Most of the “less healthy” takeaway items were consistent with the extra foods. To classify foods not identified in the extra foods list, nutrient composition data were used [43], [44]. Specifically, using food classification criteria developed by a number of Australian nutritional authorities [43], [44] takeaway items were categorised as “less healthy” if they met one or more of the following criteria:>2500 kJ of energy per serve;> 3 g of saturated fat per 100 g; <2 g of fibre per serve. Beverages classified as “less healthy” were those containing ≥300 kJ energy per serve and/or> 3 g of saturated fat per 100 g. Foods or beverages not meeting any of these criteria were considered “healthy” options. This classification resulted in nine “healthy” items and 13 “less healthy” items. “Healthy” takeaway foods comprised the first nine the first nine of the 22 items listed above, and “less healthy” takeaway foods comprised the remaining 13 items on the list. A score was subsequently calculated to characterise each participant's consumption of the 22 takeaway foods as follows: never or rarely = 0, less than once a month = 1, one to three times per month = 2, four times per month = 3, two to four times per week = 4, five to six times per week = 5, and once or more per day = 6 [45]. “Healthy” and “less healthy” takeaway food indices were created by summing the items. Each respondent's score was rescaled to range from 0 to 100 with higher scores being indicative of consuming a wider variety or greater frequency of takeaway food in the last 12 months. The reliability of the “healthy” takeaway food consumption measure was assessed in a separate test-retest sample of 100 individuals in the target age range who completed the same survey twice, four weeks apart. The ICC for the “healthy” takeaway index was 0.72 (95% CI 0.52–0.85) whereas the ICC for the “less healthy” measure was 0.69 (95% CI 0.46–0.83). According to Landis and Koch's scale of strength for reliability coefficients, these ICCs are “substantial” in magnitude [41].

### Statistical analysis

**Figure 1:**
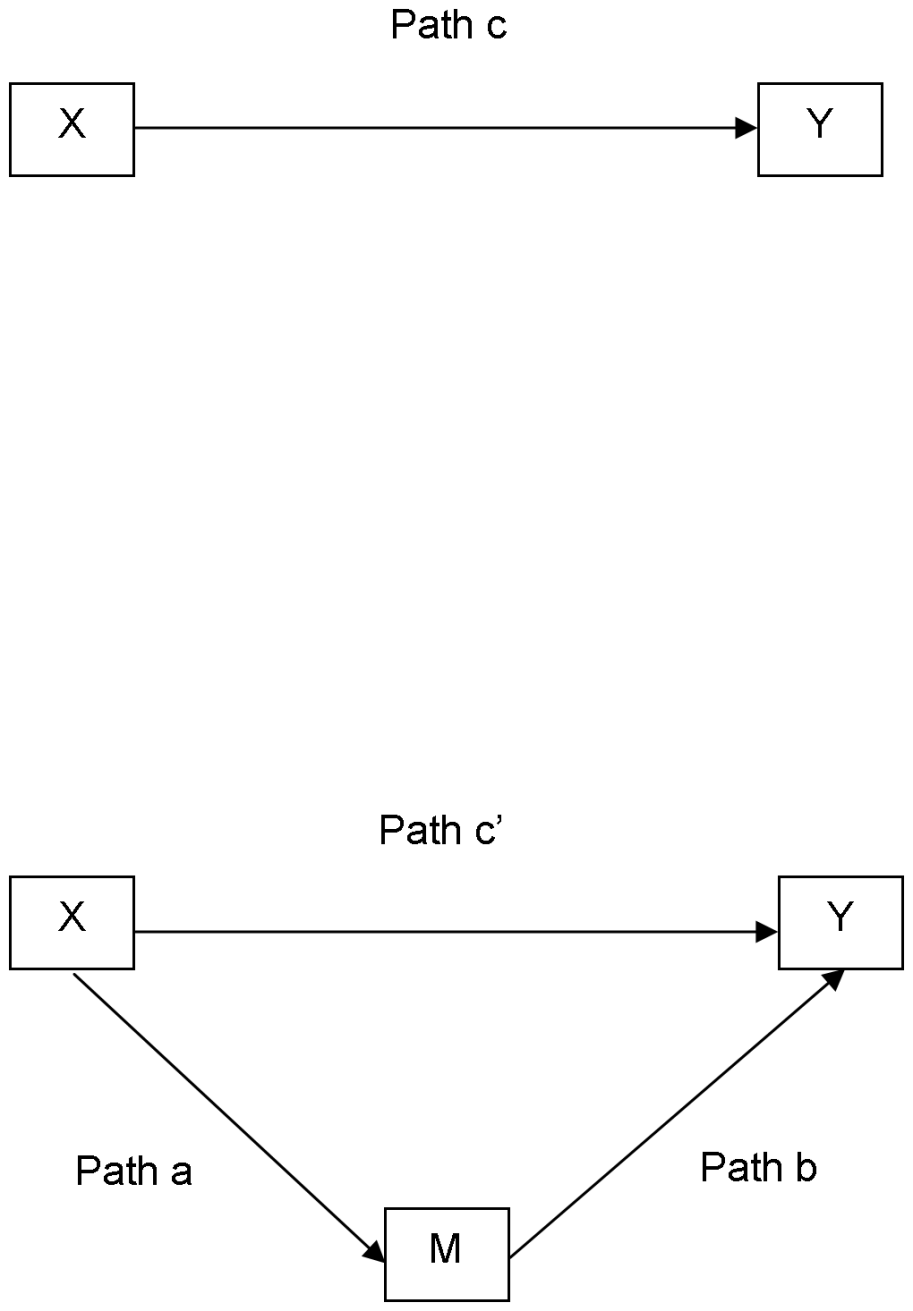
Conceptual model of the association between socioeconomic position (SEP) and takeaway food consumption and contribution of psychosocial factors to the association (adapted from [39]). X  =  the independent variable  =  SEP (education). Y  =  the outcome variable  =  takeaway food consumption (“healthy” and “less healthy”, each takeaway food type was examined separately). M  =  the proposed mediating variable  =  psychosocial factors (nutritional knowledge, belief about the diet-health relationship, perceived value of takeaway food, and takeaway foods as pleasure), each psychosocial factor was examined separately. Indirect (mediated) effect  =  a x b  =  c – c’.

Descriptive statistics were used to describe the participants' demographic and takeaway food consumption patterns. The contribution of psychosocial factors to the association between SEP and the type of takeaway food consumed was examined using a mediation model [46], [47]. A series of multiple regression models assessed a number of associations (Figure 1):

Step 1. Association between SEP and takeaway food consumption (Path c)

Step 2. Association between SEP and psychosocial factors (Path a)

Step 3. Association between psychosocial factors and takeaway food consumption (Path b)

Step 4. Association between SEP and takeaway food consumption controlling for each psychosocial factor (Path c’), and the indirect effect of SEP on takeaway food consumption through each psychosocial factor.

The mediated (indirect) effect was formally examined using a non-parametric bootstrapping procedure (n = 5000 samples) that estimated the sampling distribution of the indirect effect and the corresponding bias-corrected and accelerated 95% confidence intervals (95% CI) [46], [47]. This procedure is more statistically robust than the Sobel test [46], [47]. Indirect effects were considered significant when the 95% CI did not include zero. For all other tests, statistical significance was considered at p<0.05 (two-tailed). All models were adjusted for age and sex and the highest education group was the referent category. All analyses were performed in SAS (version 9.2, SAS Institute Inc., Cary, NC, USA).

## Results

### Exclusion of participants

Of the 903 questionnaires that were returned, missing or inadequate information was identified for age (n = 16, 1.8%), education (n = 19, 2.1%), and “healthy” and “less healthy” takeaway food indices (n = 10, 1.1%). In total, the number (%) of missing information on the psychosocial factors ranged from 5 (0.6%) to 60 (6.6%) (median n = 13, 1.4%). Participants with missing information on these variables, as well as those who reported never consuming takeaway food, were excluded from all analyses. The resultant final analytical sample was N = 801.

Table 2 shows the socio-demographic characteristics of the included and excluded participants. Over half of the study participants were females (58.8%) and the mean age was 43.8 years (SD 11.6). Compared with those who were excluded from the analyses, retained participants were younger (p<0.001) and more educated (p = 0.002).

**Table 2:** Characteristics of participants and their takeaway food consumption scores.

	Total (N = 801)	Excluded (n = 102)	Census†
**Sex (%)** ‡			
Males	41.2	37.3	49.2
Females	58.8	62.7	50.8
**Age (years) [mean (sd)]** §	43.8 (11.0)	48.2 (11.4)*	42.7 (11.0)
**Education (%)** ‡			
Bachelor degree or higher	36.5	21.7*	28.7
Diploma	12.4	10.8	10.0
Vocational	18.6	14.5	19.0
No post-school qualifications	32.6	53.0	42.3|
**Healthy takeaway food** ¶	13.2 (0.0, 73.3)	13.2 (0.0, 56.4)	
**Less healthy takeaway food** ¶	12.8 (0.0, 88.3)	12.8 (0.0, 42.2)	

† Compared with 2006 Census data (ABS, 2010).

‡ Chi-square was used to assess the differences between included and excluded participants.

§ t-test was used to assess the differences between included and excluded participants.

| People who answered “not applicable” to non-school qualifications.

¶ Median (minimum, maximum). Healthy and less healthy takeaway food consumption indices ranged from 0 to 100 with higher scores indicating a wider variety or greater frequency of consumption. Mann-Whitney U-test was used to assess the differences between included and excluded participants.

* p<0.001: statistically significant difference in age and education between the analytic sample and excluded participants.

### Association between education and takeaway food consumption (Path c)

**Figure 2:**
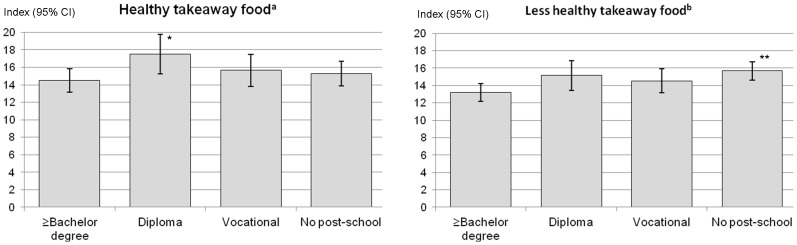
The average “healthy” and “less healthy” takeaway food consumption indices by education. The mean consumption values with their 95% confidence intervals. * p<0.05, **p<0.01 compared with participants with a bachelor degree or higher. All analyses adjusted for age and sex. ^a^ The “healthy” takeaway food consumption index (ranged from 0 to 100; mean 15.3, SD 11.7), with high score indicating a wide variety or greater frequency of consumption. *P*-for trend  = 0.512. ^b^ The “less healthy” takeaway food consumption index (ranged from 0 to 100; mean 14.0, SD 9.3), with high score indicating a wide variety or greater frequency of consumption. *P*-for trend  = 0.004.

Education was associated with type of takeaway food consumed (Figure 2). Compared with participants who had a bachelor degree or higher, those with a diploma level of education were more likely to report that they consumed “healthy” takeaway food (β = 3.02, p = 0.023). The consumption level of participants with no post-school qualifications and vocational education were also higher than that of bachelor degree or higher; however, the difference was not significant (all p>0.05). For “less healthy” takeaway food, those with no post-school qualifications scored significantly higher than participants who had a bachelor degree or higher (β = 2.38, p = 0.002). Participants with diploma level of education were also more likely to report that they consumed “less healthy” takeaway food; however, the difference was borderline significant (β = 1.99, p = 0.052).

### Association between education and psychosocial factors (Path a)

There were significant associations between education and diet and health-related beliefs and nutritional knowledge (Table 3). Compared with participants with a bachelor degree or higher, those with a vocational level of education (β = −0.28, p = 0.005) and no post-school qualifications (β = −0.31, p<0.001) were less likely to believe in the diet-health relationship and more likely to have low nutritional knowledge (vocational: β = −0.98, p<0.001; no post school qualifications β = −1.33, p<0.001). There were no education differences in the perceived value of takeaway food and the perception of takeaway food as pleasure.

**Table 3:** Associations between education level and beliefs, perceptions and knowledge about diet, health, and takeaway food†.

(N = 801)	β (SE)‡	*p*-value
**Belief about the diet-health relationship**		
Bachelor degree or higher	Reference§	–
Diploma	−0.09 (0.11)	0.435
Vocational	−0.28 (0.10)	0.005
No post-school qualifications	−0.31 (0.08)	<0.001
**Perceived values of takeaway food**		
Bachelor degree or higher	Reference§	–
Diploma	−0.04 (0.12)	0.735
Vocational	−0.12 (0.10)	0.222
No post-school qualifications	0.05 (0.09)	0.550
**Takeaway food as pleasure**		
Bachelor degree or higher	Reference§	–
Diploma	0.07 (0.12)	0.554
Vocational	−0.06 (0.10)	0.538
No post-school qualifications	−0.01 (0.09)	0.903
**Nutritional knowledge**		
Bachelor degree or higher	Reference§	–
Diploma	−0.42 (0.31)	0.178
Vocational	−0.98 (0.27)	<0.001
No post-school qualifications	−1.33 (0.23)	<0.001

† These analyses examine the association between the independent variable (education) and each mediating variable (psychosocial factor): see Figure 1, Path a.

‡ All analyses are adjusted for age and sex.

§ The regression coefficients quantify the absolute difference between the reference category and the other education categories in their mean scores on each of the psychosocial measures.

### Association between psychosocial factors and takeaway food consumption (Path b)

The majority of psychosocial factors were significantly associated with the consumption of takeaway food (Table 4). A high level of “healthy” takeaway food consumption was significantly associated with the perception that takeaway food is value for money (β = 1.46, p<0.001) or lower nutritional knowledge (β = −0.55, p<0.001). Belief in the diet-health relationship and the perception of takeaway food as pleasure was not associated with “healthy” takeaway food consumption. A high “less healthy” takeaway food score was observed among those who had a weaker belief in the diet-health relationship (β = −1.10, p<0.001), and those who perceived that takeaway food was value for money (β = 1.52, p<0.001). Participants who perceived takeaway food a pleasure (β = 1.39, p<0.001) and those who had lower nutritional knowledge (β = −0.45, p<0.001) were also significantly more likely to consume “less healthy” takeaway food.

**Table 4:** Associations between psychosocial factors and takeaway food consumption† ^,^ ‡.

(N = 801)	Healthy takeaway food§	p-value	Less healthy takeaway food|	p-value
**Belief about the diet-health relationship**	−0.61	0.139	−1.10	<0.001
**Perceived value of takeaway food**	1.46	<0.001	1.52	<0.001
**Takeaway food as pleasure**	0.12	0.765	1.39	<0.001
**Nutritional knowledge**	−0.55	<0.001	−0.45	<0.001

† These analyses examine the association between the mediating variables (psychosocial factors) and the outcome variables (takeaway consumption): see Figure 1, Path b.

‡ All analyses adjusted for age, sex, and education.

§ The healthy takeaway food consumption index ranged from 0 to 100 (median 13.2), with higher scores indicating a wider variety or greater frequency of consumption.

| The less healthy takeaway food consumption index ranged from 0 to 100 (median 12.8) with higher scores indicating a wider variety or greater frequency of consumption.

### Psychosocial contributions to the association between education and takeaway food consumption (Path c’)

Table 5 presents the association between education and takeaway food consumption (Path c), and the mediating effects of this association (Path c’). Lower educated groups consumed a high level of “healthy” takeaway food compared with those with a bachelor degree or higher.

**Table 5:** Contribution of psychosocial factors to the association between education and takeaway food consumption.

(N = 801)				Education†					
	Diploma			Vocational			No post-school qualifications		
	β (SE)‡	*p*-value§	Indirect effects (95% CI)	β (SE)‡	*p*-value§	Indirect effects (95% CI)	β (SE)‡	*p-*value§	Indirect effects (95% CI)
**“Healthy” takeaway food** ‡									
Path c	3.02 (1.33)	0.023	—	1.16 (1.15)	0.313	—	0.83 (0.98)	0.402	—
*Potential mediator*									
Belief about the diet-health relationship	2.97 (1.33)	0.026	0.05 (−0.04, 0.31)	0.99 (1.16)	0.392	0.17 (−0.02, 0.57)	0.63 (0.99)	0.523	0.19 (−0.04, 0.54)
Perceived value of takeaway food	3.08 (1.32)	0.020	−0.06 (−0.44, 0.29)	1.34 (1.15)	0.242	−0.18 (−0.53, 0.07)	0.75 (0.98)	0.443	0.07 (−0.18, 0.35)
Takeaway food as pleasure	3.02 (1.33)	0.024	0.01 (−0.07, 0.20)	1.17 (1.15)	0.310	−0.01 (−0.19, 0.07)	0.82 (0.99)	0.403	0.01 (−0.06, 0.11)
Nutritional knowledge	2.80 (1.32)	0.035	0.23 (0.00, 0.73)	0.63 (1.15)	0.586	0.53 (0.22, 1.05)*	0.10 (0.99)	0.920	0.72 (0.30, 1.30)*
**“Less healthy” takeaway food** ^|^									
Path c	1.99 (1.02)	0.052	—	1.11 (0.88)	0.209	—	2.38 (0.76)	0.002	—
*Potential mediators*									
Belief about the diet-health relationship	1.90 (1.02)	0.062	0.10 (−0.10, 0.39)	0.81 (0.88)	0.362	0.31 (0.08, 0.70)*	2.04 (0.76)	0.007	0.34 (0.12, 0.70)*
Perceived value of takeaway food	2.05 (1.01)	0.042	−0.06 (−0.44, 0.30)	1.30 (0.87)	0.137	−0.19 (−0.50, 0.06)	2.30 (0.75)	0.002	0.08 (−0.19, 0.38)
Takeaway food as pleasure	1.90 (1.01)	0.061	0.09 (−0.19, 0.44)	1.20 (0.87)	0.171	−0.09 (−0.42, 0.17)	2.36 (0.75)	0.002	0.01 (−0.24, 0.26)
Nutritional knowledge	1.80 (1.01)	0.076	0.19 (−0.01, 0.56)	0.67 (0.88)	0.448	0.44 (0.17, 0.91)*	1.78 (0.76)	0.020	0.60 (0.21, 1.08)*

† Reference category: Bachelor degree or higher.

‡ The regression coefficients quantify the absolute difference between the reference category (Bachelor degree or higher) and the other education categories in their mean scores on the “healthy” and “less healthy” takeaway consumption indexes before (Path c, Figure 1) and after adjusting for each psychosocial mediator.

§ All analyses adjusted for age and sex.

* Statistically significant indirect (mediated) effect.

After the inclusion of nutritional knowledge in the Path c model, the magnitude of the association was attenuated. However, differences between participants with a bachelor degree or higher and those with a diploma level of education remained significant (β = 2.80, p = 0.035). In general, these non-significant associations (except those with diploma level of education) were slightly reduced after the inclusion of other psychosocial factors. The indirect (mediated) effects of nutritional knowledge were significant among those with no post-school qualifications and vocational education as the 95% CI did not include zero. The largest absolute indirect effect was observed among participants with no post-school qualifications (indirect effect 0.72; 95% CI 0.30, 1.30) compared with other education groups. None of other psychosocial factors showed significant indirect effects in the consumption of “healthy” takeaway food.

Lower educated groups also scored higher for “less healthy” takeaway food (Path c). The magnitude of this baseline education difference was attenuated when diet and health-related beliefs were included in the Path c model. Among participants with no post-school education, however, the association remained significant with adjustment for the diet and health-related relationship (β = 2.04, p = 0.007). Likewise, the magnitude of the association was attenuated when nutritional knowledge was included in the Path c model. Nonetheless, differences in consumption between participants with a bachelor degree or higher and those with no post-school qualifications remained significant (β = 1.78, p = 0.020). When the variables perceived value of takeaway food and takeaway food as pleasure were included in the Path c model, the magnitude of the associations changed only slightly all levels. Significant indirect effects were observed for both diet and health-related beliefs and nutritional knowledge among those with no post-school qualifications and vocational education. The largest absolute indirect effect was observed among participants with no post-school qualifications when the mediation effect of nutritional knowledge was assessed (indirect effect 0.60; 95% CI 0.21, 1.08).

## Discussion

This study examined whether psychosocial factors contributed to the association between education and the type of takeaway food consumed. The study found that lower educated groups were more likely to consume takeaway food, especially “less healthy” options, which was similar to previous research [7], [8], [10]. The observed association between education and “healthy” takeaway food consumption was partly mediated by nutritional knowledge. Similarly, beliefs about the diet and health relationship and nutritional knowledge partly explained the education differences in “less healthy” takeaway food consumption.

Similar to this current study, previous research indicates that less educated groups have lower health considerations [48], are less likely to believe in diet-health relationships [18]–[20], and have lower nutritional knowledge [40], [49] than their more educated counterparts. Findings from this present study were also similar to studies which have shown that low nutritional knowledge and weak diet and health-related beliefs were associated with less healthy dietary patterns such as frequent fast-food consumption [20], [24], [50]. These findings suggest that intervention programs focusing on cognitive factors, especially nutritional knowledge, are likely to be important in reducing socioeconomic differences in takeaway food consumption, particularly “less healthy” options.

While nutritional knowledge and/or belief in the diet-health relationship significantly mediated the association between education and takeaway food consumption, these psychosocial factors did not completely explain the relationship. An individual's food choice is complex and is influenced by numerous factors [51] such as where the foods are to be consumed (work, leisure or home), other contexts (e.g. alone or in the presence of others) [52], culture, marketing [53] and cost [7]. Perception about the accessibility and availability of food has also been reported as influencing food choice [53]. It is possible that lower socioeconomic groups may have easier access to takeaway food outlets (i.e. greater number in their neighbourhood, more proximal) which may be one contributing factor to the high consumption of takeaway foods among these groups [54], [55]. Additionally, the availability of “healthy” and “less healthy” choices may also determine the type of takeaway food consumed, and may be a reason for the observed associations. For example, US and New Zealand studies have found that “less healthy” takeaway options are more readily available than “healthy” options at major fast-food outlets in these countries [56], [57]. Increasing and promoting the availability of healthier takeaway options, as well as facilitating the within-store purchase of healthy options, are likely to be important future directions of interventions. However, since takeaway foods are generally less nutritious than food prepared at home [58], efforts are also needed to improve the nutrient content of takeaway foods.

In this study, nutritional knowledge and the perception that takeaway foods are value for money were important determinants of both “healthy” and “less healthy” takeaway food consumption. However, there were some differences in the association between psychosocial factors and the type of takeaway food. Diet and health-related beliefs, and the perception that takeaway food is pleasure, influenced the consumption of “less healthy” takeaway food whereas these factors were not associated with “healthy” takeaway food consumption. It is unknown why diet and health-related beliefs were associated with the consumption of “less healthy” but not “healthy” takeaway food. The perception of takeaway food as pleasure was associated only with the consumption of “less healthy” takeaway food. This finding may be a reflection of the notion that, in general, eating situations (e.g. eating with others) changes an individuals' emotional state which may influence their decision to choose more “indulgent” or “less healthy” food types [59] rather than continuing to eat healthily. Likewise, the decision to consume “less healthy” takeaway food may also be driven by taste preference, which has been shown to be a strongly influential predictor of food consumption [7], [60].

Some limitations of this study need to be acknowledged. First, due to the cross-sectional design of this study, temporal direction of causal order (i.e. exposure variable precedes mediator, and the mediator precedes outcome) cannot be determined. Second, all data were collected by self-report and hence are prone to bias such as social desirability bias. These biases may be different according to participants' education level. Third, the classification of “healthy” and “less healthy” takeaway choices was made based on the AGHE [42] and nutrient composition criteria used in the Australian State Hospitals and schools [43], [44]. However, not all items in the “healthy” and “less healthy” takeaway food categories were necessarily healthy or unhealthy respectively as nutrient contents vary considerably within each food group [57], [61]. Fourth, the current study employed only one socioeconomic indicator: this is likely to have underestimated the total socioeconomic effect of takeaway food consumption. Further, the current study achieved a moderately high response rate; however, 36.7% of those approached did not respond. Typically, the most disadvantaged groups are more likely to be non-responders in a postal survey, and in general these groups are more likely to exhibit less healthy behaviours [62], [63]. In this present study, the lower educated groups were underrepresented compared with the Brisbane population hence the magnitude of the association between education and takeaway food consumption may have been underestimated.

In conclusion, intervention programs addressing nutrition and health-related beliefs and knowledge may contribute to a reduction in socioeconomic differences in takeaway food consumption. Nevertheless, the increasing popularity of takeaway food is likely to continue [64] regardless of a person's SEP, and this has implications for dietary quality, overweight/obesity and diet-related chronic disease for the whole population. Policies aimed at promoting healthy eating may need to be focused on improving the nutritional value of takeaway food and ensuring that healthy options are available. Future research should consider a wider array of psychosocial concepts (e.g. values, perceptions, and motivations) to identify and assess whether these contribute to our understanding of socioeconomic differences in takeaway food consumption.

## Supporting Information

Data S1 **Supporting data.**
(DAT)

## References

[1] Kawachi I, Kennedy BP (1999) Income inequality and health: pathways and mechanisms. Health Serv Res 34: 215–227. 10199670 PMC1088996

[2] Mackenbach JP, Kunst AE (1997) Measuring the magnitude of socio-economic inequalities in health: An overview of available measures illustrated with two examples from Europe. Soc Sci Med 44: 757–771. 9080560 10.1016/s0277-9536(96)00073-1

[3] Davey Smith G, Brunner E (1997) Socio-economic differentials in health: the role of nutrition. Proc Nutr Soc 56: 75–90. 9168522 10.1079/pns19970011

[4] James WPT, Nelson M, Ralph A, Leather S (1997) The contribution of nutrition to inequalities in health. Br Med J 314: 1545–1549. 9183207 10.1136/bmj.314.7093.1545 PMC2126753

[5] Giskes K, Turrell G, van Lenthe FJ, Brug J, Mackenbach JP (2006) A multilevel study of socio-economic inequalities in food choice behaviour and dietary intake among the Dutch population: the GLOBE study. Public Health Nutr 9: 75–83. 16480537 10.1079/phn2005758

[6] Turrell G, Hewitt B, Patterson C, Oldenburg B, Gould T (2002) Socioeconomic differences in food purchasing behaviour and suggested implications for diet-related health promotion. J Hum Nutr Diet 15: 355–364. 12270016 10.1046/j.1365-277x.2002.00384.x

[7] Blanck HM, Yaroch AL, Atienza AA, Yi SL, Zhang Jian, et al (2009) Factors influencing lunchtime food choices among working Americans. Health Educ Behav 36: 289–301. 17602103 10.1177/1090198107303308

[8] Glanz K, Basil M, Maibach E, Goldberg J, Snyder D (1998) Why Americans eat what they do: taste, nutrition, cost, convenience, and weight control concerns as influences on food consumption. J Am Diet Assoc 98: 1118–1126. 9787717 10.1016/S0002-8223(98)00260-0

[9] Miura K, Giskes K, Turrell G (2012) Socio-economic differences in takeaway food consumption among adults. Public Health Nutr 15: 218–226. 21740620 10.1017/S136898001100139X

[10] Thornton LE, Bentley RJ, Kavanagh AM (2011) Individual and area-level socioeconomic associations with fast food purchasing. J Epidemiol Community Health 65: 873–880. 20889585 10.1136/jech.2009.099614

[11] Miura K, Turrell G (2014) Reported consumption of takeaway food and its contribution to socioeconomic inequalities in body mass index. Appetite 74: 116–124. 24355907 10.1016/j.appet.2013.12.007

[12] Agardh E, Allebeck P, Hallqvist J, Moradi T, Sidorchuk A (2011) Type 2 diabetes incidence and socio-economic position: a systematic review and meta-analysis. Int J Epidemiol 40: 804–818. 21335614 10.1093/ije/dyr029

[13] Pujades-Rodriguez M, Timmis A, Stogiannis D, Rapsomaniki E, Denaxas S, et al (2014) Socioeconomic deprivation and the incidence of 12 cardiovascular diseases in 1.9 million women and men: implications for risk prediction and prevention. PLoS One 9: e104671. 25144739 10.1371/journal.pone.0104671 PMC4140710

[14] Kearney M, Kearney JM, Dunne A, Gibney MJ (2000) Sociodemographic determinants of perceived influences on food choice in a nationally representative sample of Irish adults. Public Health Nutr 3: 219–226. 10948389 10.1017/s1368980000000252

[15] Lynch JW, Kaplan GA, Salonen JT (1997) Why do poor people behave poorly? Variation in adult health behaviours and psychosocial characteristics by stages of the socioeconomic lifecourse. Soc Sci Med 44: 809–819. 9080564 10.1016/s0277-9536(96)00191-8

[16] Lê J, Dallongeville J, Wagner A, Arveiler D, Haas B, et al (2013) Attitudes toward healthy eating: a mediator of the educational level-diet relationship. Eur J Clin Nutr 67: 808–814. 23801096 10.1038/ejcn.2013.110

[17] Wang Y, Chen X (2011) How much of racial/ethnic disparities in dietary intakes, exercise, and weight status can be explained by nutrition- and health-related psychosocial factors and socioeconomic status among US adults? J Am Diet Assoc 111: 1904–1911. 22117667 10.1016/j.jada.2011.09.036 PMC3225889

[18] Girois S, Kumanyika S, Morabia A, Mauger E (2001) A comparison of knowledge and attitudes about diet and health among 35- to 75-year-old adults in the United States and Geneva, Switzerland. Am J Public Health 91: 418–424. 11236407 10.2105/ajph.91.3.418 PMC1446579

[19] Kearney M, Kelly A, Gibney MJ (1998) Attitudes toward and beliefs about nutrition and health among a nationally representative sample of Irish adults: Application of logistic regression modelling. J Nutr Educ 30: 139–148.

[20] Wardle J, Steptoe A (2003) Socioeconomic differences in attitudes and beliefs about healthy lifestyles. J Epidemiol Community Health 57: 440–443. 12775791 10.1136/jech.57.6.440 PMC1732468

[21] Beydoun MA, Wang Y (2008) Do nutrition knowledge and beliefs modify the association of socio-economic factors and diet quality among US adults? Prev Med 46: 145–153. 17698186 10.1016/j.ypmed.2007.06.016

[22] Guillaumie L, Godin G, Vezina-Im LA (2010) Psychosocial determinants of fruit and vegetable intake in adult population: a systematic review. International Journal of Behavioral Nutrition and Physical Activity 7: 12. 20181070 10.1186/1479-5868-7-12 PMC2831029

[23] Wardle J, Parmenter K, Waller J (2000) Nutrition knowledge and food intake. Appetite 34: 269–275. 10888290 10.1006/appe.1999.0311

[24] Satia JA, Galanko JA, Siega-Riz AM (2004) Eating at fast-food restaurants is associated with dietary intake, demographic, psychosocial and behavioural factors among African Americans in North Carolina. Public Health Nutr 7: 1089–1096. 15548348 10.1079/PHN2004662

[25] Aikman SN, Min KE, Graham D (2006) Food attitudes, eating behavior, and the information underlying food attitudes. Appetite 47: 111–114. 16621134 10.1016/j.appet.2006.02.004

[26] Rydell SA, Harnack LJ, Oakes JM, Story M, Jeffery RW, et al (2008) Why eat at fast-food restaurants: reported reasons among frequent consumers. J Am Diet Assoc 108: 2066–2070. 19027410 10.1016/j.jada.2008.09.008

[27] Turrell G (1998) Socioeconomic differences in food preference and their influence on healthy food purchasing choices. J Hum Nutr Diet 11: 135–149.

[28] de Boer M, McCarthy M, Cowan C, Ryan I (2004) The influence of lifestyle characteristics and beliefs about convenience food on the demand for convenience foods in the Irish market. Food Qual Prefer 15: 155–165.

[29] Mahon D, Cowan C, McCarthy M (2006) The role of attitudes, subjective norm, perceived control and habit in the consumption of ready meals and takeaways in Great Britain. Food Qual Prefer 17: 474–481.

[30] Beck ME (2007) Dinner preparation in the modern United States. Br Food J 109: 531–547.

[31] Dutton T, Turrell G, Oldenburg B (2005) Measuring socioeconomic position in population health monitoring and health research. Brisbane: Queensland University of Technology, School of Public Health.

[32] Turrell G, Hewitt B, Patterson C, Oldenburg B (2003) Measuring socio-economic position in dietary research: is choice of socio-economic indicator important? Public Health Nutr 6: 191–200. 12675962 10.1079/PHN2002416

[33] Galobardes B, Morabia A, Bernstein MS (2001) Diet and socioeconomic position: does the use of different indicators matter? Int J Epidemiol 30: 334–340. 11369739 10.1093/ije/30.2.334

[34] Dunn KI, Mohr P, Wilson CJ, Wittert GA (2011) Determinants of fast-food consumption. An application of the Theory of Planned Behaviour. Appetite 57: 349–357. 21683749 10.1016/j.appet.2011.06.004

[35] Dunn KI, Mohr PB, Wilson CJ, Wittert GA (2008) Beliefs about fast food in Australia: A qualitative analysis. Appetite 51: 331–334. 18430490 10.1016/j.appet.2008.03.003

[36] Dave JM, An LC, Jeffery RW, Ahluwalia JS (2009) Relationship of attitudes toward fast food and frequency of fast-food Intake in adults. Obesity 17: 1164–1170. 19247277 10.1038/oby.2009.26

[37] van der Horst K, Brunner TA, Siegrist M (2011) Fast food and take-away food consumption are associated with different lifestyle characteristics. J Hum Nutr Diet 24: 596–602. 21883532 10.1111/j.1365-277X.2011.01206.x

[38] Dillman DA (2000) Mail and Internet Surveys: The Tailored Design Method. New York: John Wiley & Sons, Inc.

[39] Lea EJ, Crawford D, Worsley A (2006) Public views of the benefits and barriers to the consumption of a plant-based diet. Eur J Clin Nutr 60: 828–837. 16452915 10.1038/sj.ejcn.1602387

[40] Turrell G, Kavanagh AM (2006) Socio-economic pathways to diet: modelling the association between socio-economic position and food purchasing behaviour. Public Health Nutr 9: 375–383. 16684390 10.1079/phn2006850

[41] Landis JR, Koch GG (1977) The measurement of observer agreement for categorical data. Biometrics 33: 159–174. 843571

[42] The Commonwealth Department of Health and Family Services (1998) The Australian Guide to Healthy Eating: Background Information for Nutrition Educators: Department of Health and Family Services.

[43] New South Wales Health and New South Wales Department of Education and Training (2006) Fresh Tastes at School: NSW Healthy School Canteen Strategy Canteen Menu Planning Guide. 2nd ed: New South Wales Health and New South Wales Department of Education and Training.

[44] Queensland Health (2007) A Better Choice Healthy Food and Drink Supply Strategy for Queensland Health Facilities. Queensland Health.

[45] Miura K, Giskes K, Turrell G (2011) Contribution of take-out food consumption to socioeconomic differences in fruit and vegetable Intake: a mediation analysis. J Am Diet Assoc 111: 1556–1562. 21963023 10.1016/j.jada.2011.07.009

[46] Preacher KJ, Hayes A (2004) SPSS and SAS procedures for estimating indirect effects in simple mediation models. Behav Res Methods Instrum Comput 36: 717–731. 15641418 10.3758/bf03206553

[47] Hayes AF (2013) Introduction to Mediation, Moderation, and Conditional Process Analysis: A Regression-Based Approach. New York: Guilford Press.

[48] Ball K, Crawford D, Mishra G (2006) Socio-economic inequalities in women's fruit and vegetable intakes: a multilevel study of individual, social and environmental mediators. Public Health Nutr 9: 623–630. 16923294 10.1079/phn2005897

[49] Parmenter K, Waller J, Wardle J (2000) Demographic variation in nutrition knowledge in England. Health Educ Res 15: 163–174. 10751375 10.1093/her/15.2.163 PMC4344545

[50] Beydoun MA, Powell LM, Wang Y (2008) Reduced away-from-home food expenditure and better nutrition knowledge and belief can improve quality of dietary intake among US adults. Public Health Nutr 12: 369–381. 18426638 10.1017/S1368980008002140

[51] Conner M, Armitage CJ (2006) Social Psychological Models of Food Choice. In: Shepherd R, Raats M, editors. The Psychology of Food Choice. Wallingford, Oxfordshire, UK: CABI Press.

[52] Devine CM (2005) A life course perspective: understanding food choices in time, social location, and history. J Nutr Educ Behav 37: 121–128. 15904575 10.1016/s1499-4046(06)60266-2

[53] Shepherd R (1999) Social determinants of food choice. Proc Nutr Soc 58: 807–812. 10817147 10.1017/s0029665199001093

[54] Burns CM, Inglis AD (2007) Measuring food access in Melbourne: access to healthy and fast foods by car, bus and foot in an urban municipality in Melbourne. Health Place 13: 877–885. 17470408 10.1016/j.healthplace.2007.02.005

[55] Reidpath DD, Burns C, Garrard J, Mahoney M, Townsend M (2002) An ecological study of the relationship between social and environmental determinants of obesity. Health Place 8: 141–145. 11943585 10.1016/s1353-8292(01)00028-4

[56] Saelens BE, Glanz K, Sallis JF, Frank LD (2007) Nutrition Environment Measures Study in Restaurants (NEMS-R): development and evaluation. Am J Prev Med 32: 273–281. 17383558 10.1016/j.amepre.2006.12.022

[57] Chand A, Eyles H, Ni Mhurchu C (2012) Availability and accessibility of healthier options and nutrition information at New Zealand fast food restaurants. Appetite 58: 227–233. 22019449 10.1016/j.appet.2011.10.006

[58] Guthrie JF, Lin B-H, Frazao E (2002) Role of food prepared away from home in the American diet, 1977-78 versus 1994-96: changes and consequences. J Nutr Educ Behav 34: 140–150. 12047838 10.1016/s1499-4046(06)60083-3

[59] Vue H, Degeneffe D, Reicks M (2008) Need states based on eating occasions experienced by midlife women. J Nutr Educ Behav 40: 378–384. 18984495 10.1016/j.jneb.2007.09.009 PMC2610855

[60] Tuorila H, Pangborn RM (1988) Prediction of reported consumption of selected fat-containing foods. Appetite 11: 81–95. 3239966 10.1016/s0195-6663(88)80008-4

[61] Dunford E, Webster J, Barzi F, Neal B (2010) Nutrient content of products served by leading Australian fast food chains. Appetite 55: 484–489. 20816711 10.1016/j.appet.2010.08.015

[62] Batty G, Gale C (2009) Impact of resurvey non-response on the associations between baseline risk factors and cardiovascular disease mortality: prospective cohort study. J Epidemiol Community Health 63: 952–955. 19605367 10.1136/jech.2008.086892

[63] Tolonen H, Dobson A, Kulathinal S, the WHO MONICA Project (2005) Effect on trend estimates of the difference between survey respondents and non-respondents: results from 27 populations in the WHO MONICA Project. Eur J Epidemiol 20: 887–898. 16284866 10.1007/s10654-005-2672-5

[64] Jaworowska A, Blackham T, Davies IG, Stevenson L (2013) Nutritional challenges and health implications of takeaway and fast food. Nutr Rev 71: 310–318. 23590707 10.1111/nure.12031

